# Reference Intervals (RIs) of the Bone Turnover Markers (BTMs) in Children and Adolescents: A Proposal for Effective Use

**DOI:** 10.3390/biomedicines13010034

**Published:** 2024-12-27

**Authors:** Vincenzo Brescia, Roberto Lovero, Antonietta Fontana, Roberta Zerlotin, Silvia Concetta Colucci, Maria Grano, Angela Pia Cazzolla, Francesca Di Serio, Vito Crincoli, Maria Felicia Faienza

**Affiliations:** 1 Clinical Pathology Unit, AOU Policlinico Consorziale di Bari-Ospedale Giovanni XXIII, 70124 Bari, Italy; vincenzo.brescia@policlinico.ba.it (V.B.); roberto.lovero@uniba.it (R.L.); antonietta.fontana@policlinico.ba.it (A.F.); francesca.diserio@policlinico.ba.it (F.D.S.); 2 Department of Precision and Regenerative Medicine and Ionian Area, University of Bari, 70124 Bari, Italy; roberta.zerlotin@uniba.it (R.Z.); maria.grano@uniba.it (M.G.); 3 Department of Translational Biomedicine and Neuroscience, University of Bari, 70124 Bari, Italy; silviaconcetta.colucci@uniba.it; 4 Department of Clinical and Experimental Medicine, Università degli Studi di Foggia, 71122 Foggia, Italy; 5 Interdisciplinary Department of Medicine, University of Bari Aldo Moro, Piazza G. Cesare 11, 70124 Bari, Italy; vito.crincoli@uniba.it; 6 Pediatric Unit, Department of Precision and Regenerative Medicine and Ionian Area (DiMePre-J), Medical School, University of Bari “Aldo Moro”, Piazza G. Cesare 11, 70124 Bari, Italy; mariafelicia.faienza@uniba.it

**Keywords:** adolescents, biostatistics, bone metabolism/diseases, children reference intervals

## Abstract

Background/Objectives: Bone turnover markers (BTMs) can provide information on the bone growth of apparently healthy children and adolescents or useful results in the diagnosis and monitoring of the disease condition, comparing them with appropriate reference intervals (RIs). The aim of this study was to establish the RI for the BTM [specific bone alkaline phosphatase (BALP), carboxy-terminal cross-linked collagen type I telopeptide (CTX), N-terminal propeptide pro-collagen type I (PINP), osteocalcin (OC), resistant to acid tartrate phosphatase isoform 5b (TRAcP-5b)] on serum samples from children and adolescents. Method: 202 samples from children and adolescents (ages 1–18 years) (51.48% male), considered apparently healthy. The biomarker was analyzed on automatic immunometric equipment (TGSTA Technogenetics) and the IDS-iSYS automated system kits The RI of the studied parameters was calculated according to CLSI Guideline C28-A3 with stratification by age and sex. Evaluation of the distribution of values and the meaning of the biomarker concentrations were used to calculate general and specific RI for an age group. Results: BTM concentrations vary with pubertal growth. The pattern of change differs for each bone marker. General and age-specific RI were calculated: 1–14 years, 15–18 years for BALP and CTX; 1–13 years, 14–18 years for Oc and PINP and 1–12 years, 13–18 years for TRAcP. Discussion and Conclusions: Concentrations for biomarker studied vary with age and gender. The proof of concentrations with insignificant changes until puberty led to identification of two groups of RI relating to the covariables (age and sex) for each biomarker.

## 1. Introduction

Bone tissue has high metabolic activity throughout life due to its constant remodeling. This remodeling occurs in an orderly process in which resorption is followed by formation. The cells that participate in and promote this process are osteoclasts, responsible of bone matrix, osteoblasts, in charge of the synthesis of matrix components and osteocytes, the long-lived elements acting as mechanosensors and regulators of bone remodeling through their cross-talk with osteoclasts and osteoblasts. Other cells that are recently believed to be involved in bone remodeling processes are macrophages [1].

Osteoblasts secrete bone matrix proteins, with formation of the organic bone matrix, or osteoid, which subsequently mineralizes [2]. Bone turnover markers (BTM) reflect the work of osteoblasts and osteoclasts. The production of osteoid by osteoblasts is reflected in the production of non-collagenous markers [bone alkaline phosphatase (BALP) and osteocalcin (OC)] and collagenous markers (procollagen I N-propeptide (PINP)) [3]. Type I collagen is the most abundant collagen in connective tissue, but its overall synthesis is highest in bone, where it forms the majority of the tissue’s organic matrix. Its precursor, procollagen I, is synthesized by osteoblasts, and the terminal propeptides of the molecule are cleaved extracellularly [4]. The removal of the organic bone matrix of the bone following enzymatic digestion is responsible for the bone resorption markers through the production of fragments of the degradation of type I collagen (N- and C-telopeptides of type I collagen, or NTX and CTX) and by release of the enzyme tartrate-resistant acid phosphatase type 5 b (TRAcP5b) [5]. The bone mineralization phase depends on adequate calcium and phosphate intake. In vitamin D deficiency, calcium and phosphate absorption is decreased, resulting in decreased levels of these constituents essential for bone mineralization [4].

Children have an approximately higher remodeling rate than adults [6]. During the growth of the child, the bone also changes shape, modeling, the main process that leads to bone acquisition without tissue being reabsorbed previously. A great contribution to bone growth is given by the growth plate formed mainly by type II collagen; this is reabsorbed, replaced by type I collagen and mineralized to form bone tissue. The behavior and concentrations of serum levels of bone metabolism markers in children is more complex than in adults. In fact, in adults, osteoblasts and osteoclasts are mostly dedicated to bone remodeling while in children bone growth cells also participate, which involve processes other than remodeling [7,8].

Bone growth, modeling, and remodeling result in higher bone turnover marker levels in childhood than in adults [9]. The onset of puberty is a period of high bone turnover and is associated with the closure of the growth plates, mediated by sexual hormones, with arrest of linear growth, which stops at around 14 years of age in girls and 16 in boys [10]. However, in some subjects, the growth plate remains open until the age of 30; this is why levels of bone turnover markers may still have high concentrations in young adults [11].

Clinical interpretation of bone turnover marker results in children and adolescents needing to take into consideration not only remodeling, modeling, growth, and pubertal status [12] but also the complexity of metabolic pathways that may be influenced and altered by pathological conditions. There are various genetic diseases, some chronic pathologies, or therapy-induced effects that can alter bone metabolism and therefore could benefit from the measurements of bone turnover markers. BTM may prove useful in monitoring the biological response to treatment and in evaluating the state of individual bone turnover in various pathologies. The BTM dosage could benefit in Paget’s disease [13], renal osteodystrophy, primary osteoporosis or secondary to systemic diseases, the use of medicines such as glucocorticoids [14], and malignant bone tumors, among the most common tumors among adolescents [15]. In fact, they are able to provide a dynamic picture of the change, both in the bone growth of apparently healthy subjects and in the diagnosis and monitoring of the disease condition, probably more effectively than a static evaluation obtained using dual energy X-ray absorptiometry techniques and quantitative computed tomography [16,17]. Only in certain clinical situations, as sometimes observed in severe forms of osteogenesis imperfecta (OI) where accelerated remodeling is coupled with slow growth, bone markers may not be as accurate [18].

In order for biochemical markers of bone turnover to be widely used in pediatric clinical practice, as happens, for example, in post-menopausal women to monitor osteoporosis [19], it is necessary that the results of the determinations are evaluated by comparing them with suitable reference intervals (RIs). The RIs to be used must be obtained after checking the relationships between bone formation/turnover markers, considering the age/sex variables in subjects with similar lifestyles, belonging to similar geographical areas [20]. Furthermore, reference limits must be updated to adapt to the latest laboratory methods, thus reducing differences related to analytical variability [21]. Therefore, due to differences in population, geography, lifestyle, and epidemiological manifestations of disease and analytical methods used, RIs imported from documents may not always be applicable [22].

The aim of this work was to establish reference values of bone markers of formation [bone alkaline phosphatase (BALP), osteocalcin (OC), and procollagen type I N propeptide (PINP)] and resorption [C-terminal collagen telopeptide type I (CTX), resistant to acid tartrate phosphatase isoform 5b (TRAcP-5b)] determined on serum samples from a population of children and young people under the age of 18, residing in Italy, using an immunometric method on automated instrumentation.

## 2. Materials and Methods

### 2.1. Study Subjects

This observational study was approved by ethical committees of the Policlinic University Hospital of Bari (Biomarkers of Bone Metabolism; study number 38359/COMET/BMOPed). The parents of the apparently healthy children enrolled signed the informed consent. This study was conducted in accordance with the principles of the Declaration of Helsinki and the International Conference on Harmonization Guidelines for Good Clinical Practice.

### 2.2. Inclusion Criteria

At the time of blood sampling, general health information was collected and a history of pathologies and the use of prescribed medications and vitamins or other supplements was taken. Participants’ height and weight were measured by trained personnel to the nearest 0.1 kg and 0.1 cm; measurement followed standard procedures in pediatrics. The subjects included in this study were aged >1 year and <18 years, with a normal physical work-up (weight, height, nutritional status, body mass index (BMI) within limits, and gonadal/sexual status). Samples with serum levels of creatinine, azotemia, sodium, potassium, calcium, phosphate, glycemia, liver enzymes, TSH, FreeT4, blood cell counts, albuminemia, iron, transferrin, ferritin, and C-reactive protein were considered normal and deemed suitable, with values falling within the reference limits adopted in the laboratory. Individuals who had performed intense physical exertion and had a recent gastrointestinal disease were excluded from this study as well as those with diabetes mellitus, chronic kidney disease, chronic liver disease, metabolic or hereditary bone diseases, collagen disease, endocrine disease that could have affected bone mass, neurological or musculoskeletal diseases, or severe pathology requiring hospitalization and surgical procedures in the previous six months; bone fracture within 1 year; or pregnancy or other clinical and/or metabolic conditions that could have changed the calcium–phosphate balance. Furthermore excluded: children and young adults with abnormal laboratory results and/or with vitamin D deficiency (<20 ng/mL) who had received any form of calcium and/or vitamin D supplementation and subjects under the age of 1 year for variability in bone biomarker concentrations reported in the first months of age [23].

### 2.3. Sample Collection and Storage

Blood samples were collected between January 2023 and March 2024. Fasting and early morning samples were performed in sterile vacutainers for serum samples. The samples were left to coagulate for at least 30–60 min from the time of venous sampling; after retraction of the clot, the blood samples were centrifuged at 3000 rpm for 10 min to physically separate the serum from the cellular component of the blood. Samples with potential interference from hemolysis (hemoglobin > 500 mg/dL), jaundice (conjugated and unconjugated bilirubin above 20 mg/dL), and chylosity (triglyceride concentrations > 1000 mg/dL) assessed using the HIL method [Dimension VISTA 1500 instrumentation (Siemens, Munich, Germany)]. The serum of the samples deemed suitable based on the results of the biomarkers of organ function and metabolic state was aliquoted into polypropylene tubes for storage at −80 °C, within two hours of centrifugation time, while waiting to perform the assay of the biomarkers of bone metabolism. For each subject, 2.0 mL of serum were frozen at −80 °C while awaiting the execution of the tests. The sample was thawed at room temperature and the analyses were carried out avoiding repeated freeze–thaw cycles. Furthermore, for white blood cells (wbc) (10^3^/mL) and measurement of hemoglobin (Hgb) (g/dL) the samples were collected in tubes with EDTAK3 anticoagulant for blood count and analyzed on the same day on SYSMEX XN-1000 hematology analyzer.

### 2.4. Analyte Determination

The dosage of 25(OH)Vitamin D was performed with chemiluminescence assay using the TGSTA Technogenetics instrumentation (Technogenetics, Milan, Italy). The results of all subjects included in this study were above 20 ng/mL. Total calcium (Ca) and phosphorus (P) were assayed by spectrophotometric method using the Dimension VISTA 1500 instrument (Siemens, Munich, Germany). Serum calcium (mmol/L) had average values of 2.43 (0–2 y) (RI 2.38–2.82); 2.45 (2–5 y) (RI 2.37–2.69); 2.37 (5–19 y) (RI 2.28–2.55). Serum phosphates (mmol/L) had a mean value of 1.65 (M; 1–4 y) (RI 1.42–1.99); 1.51 (M; 5–12 y) (RI 1.29–1.84); 1.42 (M; 13–15 y) (RI 1.05–1.82); 1.38 (M; 16–19 y) (RI 0.87–1.57); 1.62 (F; 1–4 y) (RI 1.42–1.99); 1.54 (F; 5–12 y) (RI 1.29–1.84); 1.35 (F; 13–15 y) (RI 1.05–1.68); and 1.34 (F; 16–19 y) (RI 0.87–1.57). Iron, glucose, and albumin analysis was carried out using the colorimetric method; sodium and potassium were quantified using the potentiometric method; creatinine and liver enzyme (AST, ALT) quantification was carried out by enzyme method; TSH, FreeT4, and ferritin quantification was carried out by chemiluminescent method (LOCI); transferrin and C-reactive protein (CRP) quantification was carried out by nephelometric method. Renal function was assessed based on creatinine levels and glomerular filtration rate estimated with the estimated glomerular filtration rate eGFR (mL/min/1.73 m^2^) (acceptability limit > 90) and with the Schwartz equation [k × height (cm)/serum creatinine], with k values different by age and sex (k = 0.55 for children younger than 12 years; k = 0.7 children ≥ 13 years), All of the following analytes were assayed on Dimension VISTA 1500 instrumentation (Siemens, Munich, Germany). The analytical quality of all analytes was monitored through the use of suitable internal QC and external QC instruments. The subjects included in this study presented results within the reference limits stratified by age and sex used in the laboratory.

### 2.5. Dosage of Bone Metabolism Biomarkers (BTM)

The level of BALP (μg/L), CTX (ng/mL), OC (ng/mL), PINP (μg/L), and TRAcP (U/L) in serum was evaluated with chemiluminescence technology on TGSTA analyzer Technogenetics (Technogenetics, Milan, Italy), using IDS-iSYS Multi-Discipline Automated System kits. The method involves the use of two monoclonal antibodies specific for the molecule to be assayed linked to biotin and acridinium. The luminescence emitted by the acridinium labeling is directly proportional to the concentration of the biomarker in the sample under examination.

Analytical measurements of BALP (μg/L), CTX (ng/mL), OC (ng/mL), PINP (μg/L), and TRAcP (U/L) in serum were performed according to the supplier’s instructions regarding preventive maintenance, initiation of functional checks, calibrations, and execution of analytical quality controls. In particular, materials for internal quality control (levels low, intermediate, and high) supplied by the manufacturing company were used and analyzed in each assay to monitor analytical precision (CV%). Based on different concentrations, the accuracy of BALP was between 4.2% and 5.0%; of CTX between 4.0% and 8.5%; of OC between 3.0% and 4.5%; of PINP between 5.0% and 5.2%; and of TRAcP between 2.6% and 5.7%. Furthermore, to verify analytical accuracy, the laboratory participated in the EQAS (External Quality Assessment Services) program of the Foundation for Pathobiochemistry and Molecular Diagnostics (Friesdorfer Straße 153, 53,175 Bonn).

#### 2.5.1. BALP Dosage

BALP is an enzyme active at an alkaline pH level that participates in the growth of hydroxyapatite crystals through the hydrolysis of pyridoxal phosphate (inhibitor of hydroxyapatite formation) [24]. BALP activity is pathologically increased in processes involving high osteoblastic activity [25] and is commonly referred to as a non-collagenous bone formation biomarker. There are several commercially available methods for measuring the mass and activity of bone ALP (BALP) [26,27,28,29,30]. Immunoassays are best suited for use in a clinical laboratory because they use BALP-specific monoclonal antibodies, are rapid to perform, easy to use, and provide reproducible data. For our study, an immunoassay reporting the mass of the enzyme was used; the method has a reportable range between 1 and 75 μg/L. The limit of detection (LoD) is 0.4 μg/L, the limit of quantification (LoQ) is 1.0 μg/L; Intralaboratory analytical CV (%) were comparable with those reported in the literature [31,32,33,34,35,36] and with those reported by the manufacturer [intra-assay CV (%) from 2.0% to 5.1%, total CV (%) from 6.6% to 9.0%].

#### 2.5.2. CTX Dosage

Currently available CTX tests provide different results, with proportional and constant distortions between different measurement ranges. The CTX kit available on the IDS-iSYS automated analyzer uses monoclonal antibodies with specificities equal to those used for Elecsys 2010 and E170 immunoassays (Roche Diagnostics, Penzberg, Germany), the first automated analyzers that measured CTX. However, the IDS-iSYS provides lower CTX values than the Roche CTX test [37]. According to the manufacturer’s report, the reportable range is 0.033 to 6.000 ng/mL, the LoD is 0.023 ng/mL, the LoQ is 0.033 ng/mL, intra-assay CV(%) 2.1% to 4.9%, and based on different analyte concentrations in the samples, the total CV (%) is from 4.7% to 8.8%.

#### 2.5.3. OC Dosage

Commercially available OC tests use different methodologies (RIA, IRMA, and ELISA), different antibodies (polyclonal or monoclonal), and different standards [38]. Circulating OC is not a single amino acid peptide but rather several fragments [39]. Tests for OC are mainly based on total OC assays performed on automated immunoassay analyzers [40]. These assays have replaced previous methods including manual ELISAs [41]. The total OC assay we use has a reportable measurement range between 2 and 200 ng/mL, LoD of 0.27 ng/mL, and LoQ of 1.57 ng/mL. The method has an intra-assay repeatability CV (%) ranging from 1.8% to 3.8% and an overall repeatability ranging from 3.7% to 9.2% [39,40].

#### 2.5.4. PINP Dosage

PINP was initially isolated from amniotic fluid, and amino acid sequencing identified the high-molecular-weight form as a homodimer of the α1 chains of PINP. Commercially available assays for measuring circulating PINP use several antibodies against the α1 chain of PINP (139, 140). PINP is present in serum either as a trimer (“intact”) or as a monomer. The automated iSYS-IDS (Immunodiagnostics System) measures only the trimeric form are called intact assays whereas assays that detect both monomeric and trimeric forms are defined as total assays (e.g., Roche PINP assay). The advantage of the intact assay appears to be its specificity for PINP and its lack of cross-reactivity with the so-called monomeric [42,43]. According to the manufacturer’s report, the reportable measurement range of the assay is 2–230 ng/mL, LoD and LoQ are <1.0 μg/L, intra-assay repeatability CV (%) is from 2,6% to 3%, and total variability is from 4.2% to 5.3%.

#### 2.5.5. TRAcP Dosage

TRAcP5b serum concentrations correlate with the number of osteoclasts. [44] Several immunoassays have been developed to quantify the isoforms and have specificity for TRAcP5b and minimal interference with the 5a form. TRAcP5b can be measured by an automated method (IDS iSYS) and a manual EIA (Nittobo Medical), both methods showing good agreement. The results of the comparison between the few immunometric methods currently available demonstrate that the harmonization of the results obtained is possible using a common calibrator [45]. According to the manufacturer’s report, the reportable measurement range of the assay is 0.6 U/L, LoD and LoQ are 0.9 U/L, intra-assay repeatability CV (%) is from 2.6% to 3%, and total variability is from 4.2% to 5.3%.

### 2.6. Statistical Analyses

The mean and median concentrations, the standard deviation (SD), and distribution intervals [0.05 and 0.95 centiles and 95% confidence intervals (CI)] of BALP, CTX, OC, PINP, and TRAcP were calculated using standard parametric and non-parametric statistical analyses. Distribution normality was evaluated using the D’Agostino–Pearson test; the distribution was considered normal if the *p* value was greater than 0.05. To visualize the distribution of the samples and the underlying normality curve, the frequency distribution histogram (%) was used. To identify and exclude any data outliers, a statistical approach was used after partitions with normally distributed data (Tukey test). The presence of even just one value of a biomarker of bone metabolism “suspected outliers” was used as a criterion for excluding the patient from the statistical calculation. Boxplots of concentration were produced. The central box shows the values from the 25th to the 75th quartile, the central line is the median, and the horizontal lines are the extension from the minimum to the maximum value (range of the distribution for single biomarker) for a visual interpretation of numerical data and for showing the number of concentrations that fall outside the specified range of values (outside value). The reference interval for the studied parameters was calculated as the interval in which the central values of 90% of apparently healthy subjects are located. According to CLSI C28-A3, a double-sided reference interval was calculated; therefore, 2 limits of normality were produced: a lower limit in which 5% of the healthy subjects included in this study have lower values, and an upper limit of normality in of which 5% of healthy subjects have higher values. Calculating a reference interval using the robust method involves an iterative process, in which actual observations are underweighted based on their distance from the central tendency of the sample (CLSI 2008) [23]. The robust method was used as an alternative to the percentile method since its use is recommended for sample sizes less than 120. The traditional summary statistic was integrated by the visualization of the density trace of the values in total and as a function of sex reported in a violin plots. The graph shows the distribution of data in the form of points and the corresponding distribution, which is wider in the sections where there are more data and narrower in the sections where there are fewer data. The median value and CI of the median is drawn inside the violin graph. The measurements are modeled on age (8 age groups) using weighted polynomial regression, which provides the mean of the measurements as a function of age (mean age) values at 0.05 and 0.95 centiles stratified according to the different age groups and sex (male and female) to identify the differences to be used for the calculation of RI. The apparently healthy subjects recruited into the study were arbitrarily divided into three age groups (1) 1–6 years, (2) 7–12 years, and (3) 13–18 years [46]. The Mann–Whitney U test was used to evaluate whether the median biomarker concentrations stratified across the three age groups and by sex came from the same population (statistically significant *p*-value < 0.05). Multiple comparison procedures (pairwise comparison) were used in an ANOVA test to compare the means obtained in the subgroups stratified by age (significant difference between measurements *p* < 0.05). To visualize the distribution and dispersion of the measurements versus age a scatter plot was used with the calculated mean (central line) and centile curves, a graph showing the z-scores calculated as [(x − reference value)/SD] plotted according to age (suitability criterion less than 5% of cases above or below the line corresponding to z = +/−1.645) was used to identify significant variations in biomarker concentrations as a function of age. The evaluations of the distribution of values in the scatter plot (centile curves), the trend (central line) of the z-scores, and the significance of the biomarker concentrations in the three subgroups of subjects evaluated was used to recalculate the reference value in an age range with minimal variations in concentration. The MedCalc^®^ (11,6,1,0) program was used for statistical analyses.

## 3. Results

**Figure 1:**
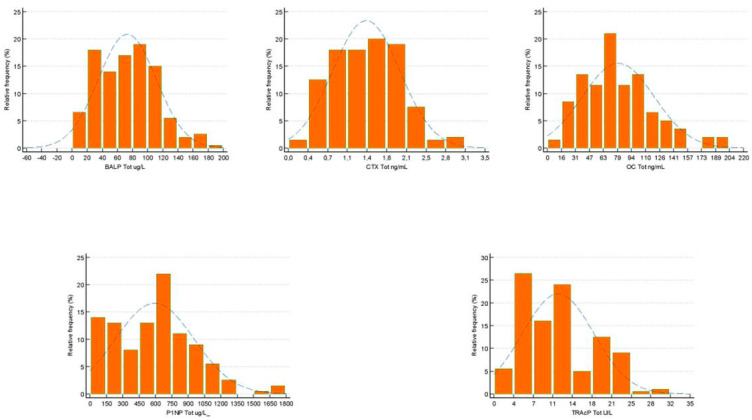
Frequency histogram of BALP (ug/L), CTX (ng/mL), OC (ng/mL), PINP (ug/L), and TRAcP (U/L) concentration in pediatric subjects evaluated.

**Figure 2:**
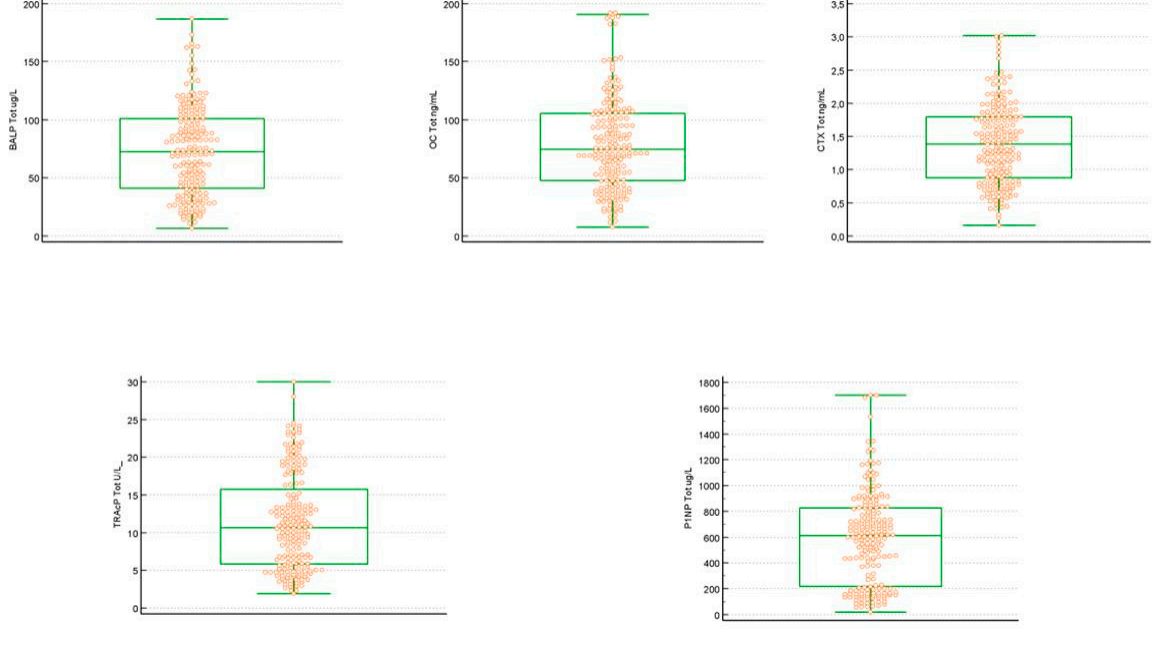
Boxplot of BALP (μg/L), CTX (ng/mL), OC (ng/mL), PINP (μg/L), and TRAcP (U/L) concentration.

A total of 202 samples were considered evaluable, according to CLSI C28-A3, which indicates a minimum number of 120 objects to be included in studies for the evaluation of reference interval (RI). A descriptive statistical analysis of the distribution of the samples after the exclusion of the outlier values is reported in Table 1. In the group of subjects evaluated, 104 (51.48%) individuals were male and 98 (48.52%) were female. All samples evaluated had concentrations above the LoD. The distribution of biomarkers evaluated with the D’Agostino–Pearson test was non-normal (*p* is less than 0.05). The data obtained were confirmed by the analysis of frequency distribution histogram (%) and visual comparison of the observed frequency distribution with the underlying theoretical normality curve (Figure 1). The boxplots of the samples selected for the statistical evaluation did not highlight the presence of suspected outliers (Figure 2). The data obtained from the weighted polynomial regression are reported in Table 2.

### 3.1. Calculation of Reference Limit

BALP: The lower limit was 14.59 μg/L in the total samples, and 12.69 μg/L and 15.84 μg/L in females and males, respectively. The upper limit was 162.28 μg/L in the total samples, and 150.71 μg/L and 167.64 μg/L in females and males, respectively.

CTX: The lower limit was 0.41 ng/mL in total samples, and 0.32 ng/mL and 0.52 ng/mL respectively in females and males. The upper limit was 2.90 ng/mL in the total samples, and 2.90 ng/mL and 2.91 ng/mL in the females and males, respectively.

OC: The lower limit was 17.81 ng/mL in the total samples, and 14.01 ng/mL and 22.74 ng/mL in females and males, respectively. The upper limit was 191.92 ng/mL in the total samples, and 189.68 ng/mL and 192.63 ng/mL in the females and males, respectively.

PINP: The lower limit was 63.60 μg/L in the total samples, and 57.76 μg/L and 86.93 μg/L in females and males, respectively. The upper limit was 1517.03 μg/L in the total samples, and 1316.31 μg/L and 1696.10 μg/L in the females and males, respectively.

TRAcP: The lower limit was 2.81 U/L in the total samples, and 2.45 U/L and 3.17 U/L in females and males, respectively. The upper limit was 24.10 U/L in the total samples, and 24.35 U/L and 25.72 U/L in females and males, respectively.

The calculation of “lower limit” and “upper limit” obtained from the samples of female subjects provided lower values than those of male subjects regardless of the bone metabolism biomarker evaluated. The data are reported in Table 3.

**Figure 3:**
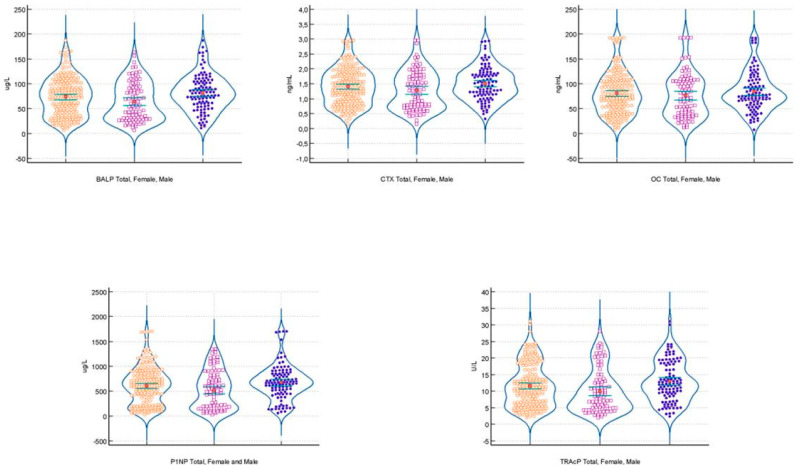
The violin plots of BALP (ug/L), CTX (ng/mL), OC (ng/mL), PINP (ug/L), and TRAcP (U/L) concentration in total (orange dots), in females (violet dots), and in males (blue dots). The median value (red dot) and CI of the median (green lines) are drawn inside the violin graph.

The violin plots of biomarker values in the pediatric subjects included for the calculation of the reference value confirms the different distribution of values in female subjects vs. males (Figure 3).

### 3.2. Stratification of Results According to Age and Gender

**Figure 4:**
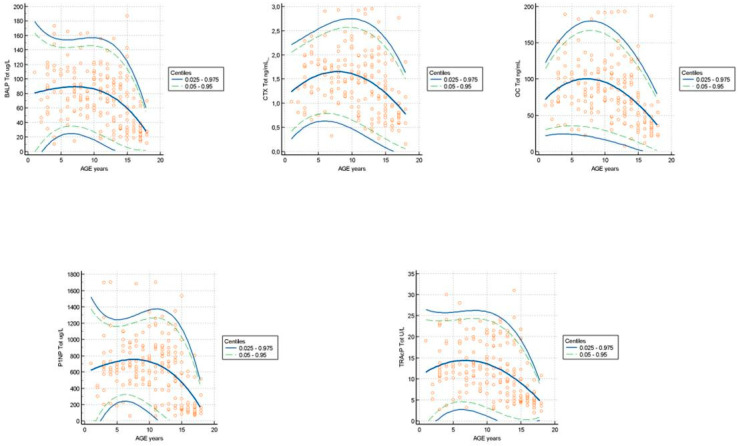
Plot of the scatter diagram of the measurements of BALP (µg/L), CTX (ng/mL), OC (ng/mL), PINP (µg/L), and TRAcP (U/L) versus age. The graph shows the calculated mean (central line) and centile curves (solid line: 0.025–0.975 centiles; broken line: 0.05–0.95 centiles) to the individual measurements.

**Figure 5:**
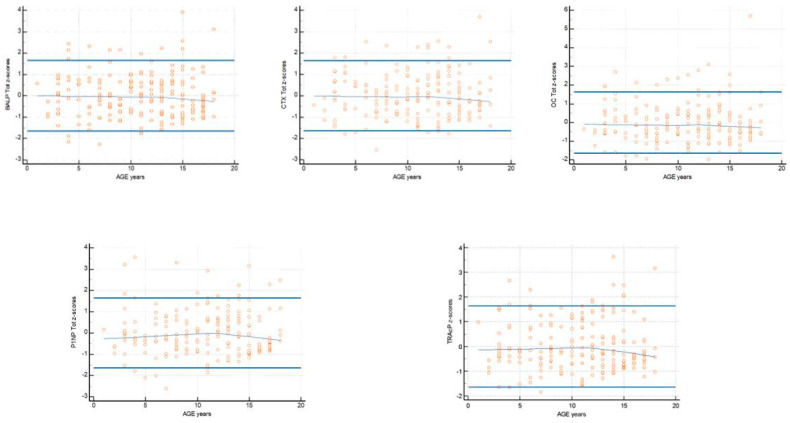
Graphs reporting the z-scores of the measurements of BALP (ug/L), CTX (ng/mL), OC (ng/mL), PINP (ug/L), and TRAcP (U/L) plotted against age. Horizontal lines are drawn at z-scores of −1.645 and 1.645; the trendline is in the center.

BALP: The data obtained from the weighted polynomial regression and reported in Table 2 highlight that the age group of 2 years presents the greatest degree of dispersion with a difference equal to 142.06 μg/L, an average of 71.03 μg/L, and the highest concentration value of 154.27 μg/L. The highest 0.05 centile value was 33.65 μg/L and the lowest was 3.03 μg/L in age groups of 8 and 16 years, respectively. The age group of 16 years showed the lowest dispersion of values with a concentration difference of 93.45 μg/L, an average of 46.72 μg/L and the lowest concentration at 0.05 centiles and 0.95 centiles of 3.03 and 96.48 μg/L. The graph of the dispersion of the measurements highlights an apparent different distribution of the BALP in the early years (age 1–5 y), no evident variation in subjects with age up to 14 years, and a progressive reduction of concentrations in the older subgroup (Figure 4). The z-scores showed a decline in the mean distribution line starting from subjects aged 14 years. The percentage of cases included between the acceptance lines corresponding to z = +/−1.645 is less than or equal to 5%. (Figure 5). The Mann–Whitney test highlighted significant differences (*p* < 0.05) between the median concentrations of BALP in the group of pediatric subjects 13–18 years vs. 1–6 years and 7–12 years. It also highlighted significant differences (*p* < 0.05) between the median BALP concentrations between female vs. male, as well as statistically significant differences in the 13–18 year age group. The test results are shown in Table 4. The significant multiple comparison procedures ANOVA test highlights that the means obtained in the subgroup stratified by age 13–18 years showed a significant difference vs. the 1–6 year and 7–12 year groups (*p* < 0.05). The test results are shown in Table 5.

*Calculation of Reference Limit age range 1–14 years*

The lower limit was 22.32 μg/L in the total samples; it was 20.56 μg/L and 24.55 μg/L, respectively, in females and males. The upper limit was 163.54 μg/L in the total samples and 155.53 μg/L and 169. 49 μg/L in females and males, respectively (Table 3).

*Calculation of Reference Limit age range 15–18 years*

The lower limit was 7.49 μg/L in the total samples, and 6.6 μg/L and 16.5 μg/L, respectively, in females and males. The upper limit was 123.00 μg/L in the total samples, and 60.6 μg/L and 130.9 μg/L in females and males, respectively (Table 3).

CTX: The data obtained from the weighted polynomial regression and reported in Table 2 highlight that the age groups of 4 years present the lowest degree of dispersion with a difference equal to 1.58 ng/mL and an average of 0.79 ng/mL. The highest concentration value of 2.56 ng/mL at 0.95 centiles was in the age group of 10 years. The maximum degree of dispersion was observed in the age group of 12 years with a difference of 1.98 ng/mL and an average of 0.99 ng/mL. In the age group of 16 years, the lowest concentration was found at 0.05 centiles and 0.95 centiles of 0.18 and 1.97 ng/mL, respectively.

The graph of the dispersion of the measurements highlights a variation distribution of the CTX in the group of subjects at age 1–14; the graph highlights a reduction in concentration in the subgroup over 15 years of age (Figure 4). The z-scores showed a decline in the mean distribution line starting from subjects aged 14 years. The percentage of cases included between the acceptance lines corresponding to z = +/−1.645 is less than 5% (Figure 5).

The Mann–Whitney test highlighted significant differences (*p* < 0.05) between the median CTX concentrations in the group of pediatric subjects 13–18 years vs. 1–6 years and 7–12 years. It also highlighted significant differences (*p* < 0.05) between the median CTX concentrations between female and male subjects overall and in the 13–18 year age group. The test results are shown in Table 4.

The significant multiple comparison procedures ANOVA test highlights that the means obtained in the subgroup stratified by age 13–18 years showed a significant difference vs. 7–12 years group (*p* > 0.05). The results of the test are reported in Table 5.

*Calculation of Reference Limit: age range 1–14 years*

The lower limit was 0.48 ng/mL in the total samples, and 0.43 ng/mL and 0.60 ng/mL, respectively, in females and males. The upper limit was 2.86 ng/mL in the total of the samples, of 2.96 ng/mL and 2.85 ng/mL in females and males, respectively (Table 3).

*Calculation of Reference Limit age range 15–18 years*

The lower limit was 0.18 ng/mL in the total samples; 0.16 ng/mL and 0.54 ng/mL, respectively, in females and males; the upper limit was 2.33 ng/mL in the total samples, and 1.44 ng/mL and 2.36 ng/mL in females and males, respectively (Table 3).

OC: The data obtained from the weighted polynomial regression and reported in Table 2 highlight that the age group of 8 years presents the highest degree of dispersion with a difference equal to 134.02 ng/mL and the highest concentration value 167.08 ng/mL at 0.95 centiles. The age group 16 years showed the lowest dispersion of values with a concentration difference of 93.48 ng/mL, with the lowest concentrations at 0.05 centiles and 0.95 centiles of 8.72 and 102.2 ng/mL. The graph of the dispersion of the measurements did not highlight an apparent different distribution of the OC in the groups of subjects at age 1–12; the graph highlights a reduction in the concentration in the subgroup over 13 years of age (Figure 4). The z-scores showed a decline in the average distribution line starting from subjects aged 13 years. The percentage of cases included between the acceptance lines corresponding to z = +/−1.645 was less than 5% (Figure 5).

The Mann–Whitney test highlighted significant differences (*p* < 0.05) between the median OC concentrations in the three different groups of pediatric subjects (1–6 years, 7–12 years, and 13–18 years) and between the concentrations medians of OC among female vs. male subjects only in the 13–18 year age group. Furthermore, there was no significant difference (*p* > 0.05) between the median OC concentrations between female vs. male subjects. The test results are shown in Table 4.

The significant multiple comparison procedures ANOVA test highlights that the means obtained in the subgroup stratified by age 7–12 years showed a significant difference vs. the 1–6 year and 13–18 year groups (*p* > 0.05). The results of the test are reported in Table 5.

*Calculation of Reference Limit: age range 1–13 years*

The lower limit was 25.33 ng/mL, and 15.21 ng/mL and 24.08 ng/mL in total samples for females and males, respectively. The upper limit was 190.07 ng/mL in the total samples and 191.83 ng/mL and 187.07 ng/mL in the females and males, respectively (Table 3).

*Calculation of Reference Limit age range 14–18 years*

The lower limit was 14.80 ng/mL in the total samples, and 12.70 ng/mL and 23.10 ng/mL, respectively, in females and males. The upper limit was 153.14 ng/mL in total of the samples and 109.0 ng/mL and 187.10 ng/mL in females and males, respectively (Table 3).

PINP: The data obtained from the weighted polynomial regression and reported in Table 2 highlight that the age group of 2 years presents the highest degree of dispersion with a difference equal to 1217.92 μg/L and the highest concentration value 1267.89 ug/L. The age group 16 years showed the lowest dispersion of values with a concentration difference of 824.77 μg/L. The lowest concentration at 0.05 centiles of 40.78 μg/L and at 0.95 centiles of 865.69 μg/L was found in the group of pediatric subjects at 14–15 years and 16–18 years, respectively.

The graph of the dispersion of the measurements highlights an apparent different distribution of the PINP in the group of subjects with age 1–10; it also highlights a progressive reduction in concentrations starting from the subgroup over 13 years of age (Figure 4). The z-scores show a decline in the mean distribution line starting from subjects aged 13 years. The percentage of cases included between the acceptance lines corresponding to z = +/−1.645 is less than or equal to 5% (Figure 5). The Mann–Whitney test highlights significant differences (*p* < 0.05) between the median concentrations of PINP in the groups of pediatric subjects 1–6 years vs. 7–12 years and 7–12 years vs. 3–18 years. It also highlights differences significant (*p* < 0.05) between the median PINP concentrations among female vs. male subjects and between the median PINP concentrations among female vs. male subjects in the 13–18 year age group. The test results are reported in Table 4.

The significant multiple comparison procedures ANOVA test highlights that the means obtained in the subgroup stratified by age 13–18 years showed a significant difference vs. 1–6 years and 7–12 years groups (*p* < 0.05). The results of the test are reported in Table 5.

*Calculation of Reference Limit age range 1–13 years*

The lower limit was 144.48 μg/L in the total samples and 143.63 μg/L and 167.09 μg/L, respectively, in the females and males. The upper limit was 1617.07 μg/L in the total of the samples and 1327.29 μg/L and 1693.04 μg/L in females and males, respectively (Table 3).

*Calculation of Reference Limit age range 14–18 years*

The lower limit was 14.80 μg/L in the total samples, and 48.55 μg/L and 83.3 μg/L, respectively, in females and males. The upper limit was 959.22 μg/L in total of the samples and 1025.0 μg/L and 1040.0 μg/L in females and males, respectively (Table 3).

TRAcP: The data obtained from the weighted polynomial regression and reported in Table 2 highlight that the age group of 2 years presents the greatest degree of dispersion with a difference equal to 22.56 U/L. The highest concentration value at 0.95 centiles of 24.32 U/L was recorded in the 8 year group of subjects; the age group of 16 years showed the lowest dispersion in values with a concentration difference of 14.45 U/L and the lowest concentration at 0.05 centiles and 0.95 centiles of 0.28 and 14.83 U/L, respectively.

The graph of the dispersion of the measurements highlighted an apparent different distribution of the TRAcP in the group of subjects at age 1–10; the graph highlights a reduction in concentrations in the subgroup over 12 years of age (Figure 4). The z-scores showed a decline in the mean distribution line starting from subjects aged 12 years. The percentage of cases included between the acceptance lines corresponding to z = +/−1.645 is less than or equal to 5% (Figure 5). The Mann–Whitney test highlighted significant differences (*p* < 0.05) between the median concentrations of TRAcP in the group of pediatric subjects 13–18 years vs. 1–6 years and 7–12 years. Furthermore, there were significant differences (*p* < 0.05) between the median concentrations of TRAcP between female vs. male subjects, especially in the 13–18 year age group. The test results are shown in Table 4.

The significant multiple comparison procedures ANOVA test highlights that the means obtained in the subgroup stratified by age 13–18 years showed a significant difference vs. 1–6 year and 7–12 year groups (*p* < 0.05). The results of the test are reported in Table 5.

*Calculation of Reference Limit age range 1–12 years*

The lower limit was 5.38 U/L in the total samples and 4.60 U/L and 6.72 U/L, respectively, in females and males. The upper limit was 24.11 U/L in the total of the samples and 24.00 U/L and 23.40 U/L in females and males, respectively (Table 3).

*Calculation of Reference Limit age range 13–18 years*

The lower limit was 2.30 U/L in the total samples, and 2.34 U/L and 1.90 U/L, respectively, in females and males. The upper limit was 23.98 U/L in total of the samples and 21.28 U/L and 30.0 U/L in females and males, respectively (Table 3).

## 4. Discussion

BTM reflect both bone formation and resorption. The extent of these processes varies across the lifespan, making age an important source of variability. All BTMs increase during growth; the increase varies between different BTMs, from 2 times higher than the average adult level for TRAcP5b to 10 times higher for BALP at mid-puberty [47]. This increase is followed by a decrease to adult levels in late puberty. The decrease is later in boys than in girls, depending on the time of puberty [12,47,48,49].

Biochemical measurements of bone turnover are essential in studying the pathophysiology of skeletal metabolism and growth. However, interpretation of the results is difficult because such measurements fluctuate depending on age, pubertal stage, growth rate, mineral accumulation, hormonal regulation, nutritional status, circadian variation, vitamin concentrations D, geographical area and lifestyle, and of the sensitivity and specificity of the analytical method [9].

All of these variables should be considered while developing reference ranges to be used to interpret BTM results in the skeletal growth phase [50,51].

The collection of samples for the measurement of bone metabolism biomarkers required a correct evaluation of the different sources of variability; age and sex are not controllable, but the state of health and physical activity of the subjects recruited for this study, the time of sampling, the method of collection and conservation of the samples, and the verification of analytical quality are controllable factors and therefore were monitored. Our study was conducted on apparently healthy children and adolescents whose health status was assessed based on anamnestic data and biochemical evaluation. It was recommended to avoid physical exercise for 48 h before sample collection as some studies report that it can cause acute changes in BTM concentrations [52,53]. The evaluation required that samples be collected at the same time of day, ideally before 11.00 a.m. and fasting because BTM show circadian variations and fluctuations in food intake [53,54,55]. To reduce pre-analytical variability in the storage phase, it was taken into account that BALP remains stable after long-term storage at −20 °C and up to three freeze–thaw cycles [56]; OC [57], TRAcP5b [58], and PINP [59,60] exhibit little or no reduction in serum stored immediately after collection at −70 °C and CTX measured in serum and stored at −30 °C is unaffected from 12 weeks of storage or repeated freezing–thawing cycles [61]. Pregnant women were excluded from the evaluation, as a significant increase in BTM is reported from the 14th week of gestation [62], as well as subjects with recent fractures (within 1 year), as it is reported in the literature that BTMs return to baseline level several months after the fracture [63]. During the years of puberty, calcium requirements increase, and the sufficiency of vitamin D (25(OH)D) is important to for allow optimal calcium absorption [64]; therefore, we used a concentration above 20 ng/as an inclusion criterion [65,66,67,68,69,70]. It was necessary to guarantee the quality of the analytical phase by verifying the precision in the execution of the tests and verifying the data with concentrations above the limit of quantification (the lowest concentration with adequate precision) [22].

In the literature, RIs of bone metabolism markers are observed based on reports of pubertal maturation in adolescents (Tanner stages) for both sexes. However, some studies highlight that Tanner stages provide an inaccurate assessment with an underestimation of pubertal stage in young girls and an overestimation in adolescent boys if staging is not performed by specifically trained medical personnel [21,71].

This systematic bias in the categorization of the Tanner stage, influenced by the qualification level of the professionals, led us to prefer a stratification based on age rather than on the assessment of pubertal maturation.

### Reference Intervals

Data on BALP highlight an increase in BALP concentrations in the first years of life, a decline in concentrations starting from the age of 14, and from significantly lower values in the age group over 16 years. The age group of 16 years showed the lowest dispersion in values with a concentration difference equal to 93.45 μg/L, concentrations lower than 0.05 centiles and 0.95 centiles, and statistically significant differences in females vs. males. When comparing our RI with the results of other work, there is a degree of homology. The same trend is observed with a small initial increase followed by a progressive decrease with pubertal age. This trend of values is found in other works despite the results being obtained with different immunoassays on automated analyzers (Roche Elecsys and Liaison XL) and on populations residing in different geographical areas (Canada and Europe) [20,72]. BALP concentrations during childhood and puberty show the same trends as PINP concentrations, with a trough during childhood and a peak in early/mid puberty [72,73,74]. Furthermore, BALP, like PINP, is closely associated with the speed of growth in height [48]. The evidence of non-significant differences in BALP concentrations in subgroups of pediatric subjects up to 14 years of age led to the decision to use a common RI up to that age. Furthermore, as stated by other authors, we believe that there is no need for gender-specific reference intervals under the age of 14 (*p* > 0.05) [74].

The trend of the distribution of PINP values highlights higher concentrations in the first years of life with a similar trend to BALP, concentrations with minimal variation up to the subgroup aged over 12 years, from lower values in the age group over 16 years, and statistically significant differences between female and male subjects in the 13–18 year age group. The z-scores confirm the bending of the average distribution line starting from subjects aged 12 years. The trend of these data is similar to that reported in other studies [20,75,76]. The pubertal peak of PINP is earlier in girls than in boys and corresponds to Tanner stages II/III of breast development in girls and Tanner stage III of genital development in boys [77]. The mean values of PINP concentrations of children aged 6 years are not different when compared with US work [78]. The upper limit of the RI of the PINP does not differ in boys and girls up to the age of 12; in this age group, the lower and upper limits of PINP are comparable with those reported in other studies [77]. It has recently been demonstrated that automated PINP methods provide harmonized results; however, the different methods of stratification of the age groups do not allow for a correct interpretation and perfect correlation of the results between the different works reported in the literature [79,80].

The expression profile of CTX during infancy differs from PINP, not showing a peak in early childhood. During childhood, CTX expression is relatively stable, increasing slightly to reach a peak in early puberty, followed by a decrease [9,20,48,81,82]. The changes in concentration in the pubertal development phase produce an increase in concentration up to 2.56 ng/mL without differences between sexes, and significantly lower values in the age group over 16 years; the reduction appears earlier in girls than in boys. The trend described presents a decline in concentrations that tends to be later than that of OC and TRAcP. Other authors have demonstrated a similar trend, with concentrations without clinically relevant variations up to the age of 9 years [48,78] and an increase associated with the pubertal growth spurt with a peak in subjects aged 10–13 years [50,83]. In consideration of the data obtained, we proposed a reference (RI) for the CTX concentration for a group of subjects stratified by age (1–14 years) and in addition to two age groups (15–18 years) stratified by sex (female and male) (Table 3). The differences found in the literature, however, could be interpreted on the basis of a different stratification by age [8], in addition to vitamin D being possible a season-related inverse relationship determinant [84,85], and BMI [86].

Our data show a trend in TRAcP values more similar to BALP than to the other BTMs. The concentrations have a trend characterized by the greater degree of dispersion of the values in childhood, a tendency towards a stability of the concentrations up to the age group 8 years, and a progressive decline in the concentrations starting in the age group 10 years and from significantly lower values in the age group 16 years. Statistically significant differences are evident between female and male subjects in the 13–18 year range. A study of Chinese children showed similar behavior: TRAcP 5b levels were high in young children of both sexes, with a tendency to gradually decrease with age (*p* < 0.001) without any significant difference between sexes (*p* = 0.682), and an increase at 12–13 and 10–11 years in males and females (*p* < 0.001) [87]. Regardless of differences related to the analytical method, the distribution of BALP and TRAcP activity in relation to age (and therefore puberty) and sex is quite comparable between different works [73]. The data obtained and the comparison with the literature suggest adopting RI lower and upper limits differentiated by sex starting from 13 years.

Data regarding OC expression profiles vary in the literature. While some have found a fairly stable pattern [88], others demonstrate that OC is elevated during childhood, declines, and subsequently peaks in puberty [9,10]. The graph of the dispersion of the measurements obtained by us highlighted a progressive and significant increase in the OC in the group of subjects at age 1–6 years; the age group of 8 years presents the greatest degree of dispersion and the highest concentration value. Starting from the age group of 12 years, there is a decline in the average distribution line and significantly lower values in the age group of 16 years. The lowest concentration at 0.05 centiles and 0.95 centiles of 8.0 ng/mL and 102.2 ng/mL was found in the group of 16–18-year-old subjects. Differences in concentrations by sex are only evident from the age of 15. Interestingly, the increase tends to be later than that of BALP and PINP and the similarity of the trend to CTX and TRAcP with a decline in concentrations starting from the age of 15 and significantly lower values in the age group over 16 years [9]. Our results highlight a good degree of similarity with other studies [20,72,77,89].

Geographic origin and ethnicity are usually key elements in establishing a valid RI. The trend in concentrations was similar to studies produced in Canada, central Europe, the United States, Brazil, and Korea; therefore, it is likely that in children geographical origin and ethnicity have a lesser influence on RI in the face of growth-related changes and growth spurts. The environmental variable could be crucial for the availability in the study area and population of sufficient levels of nutrients, vitamins, and minerals, essential elements for normal bone growth.

One of the limitations in defining the reference interval in children was finding a sufficient number of healthy subjects. Our study took into consideration what was recommended by the CLSI working group to generate reliable results even in small sample sizes [89,90], and the removal of anomalous “outlier” values was an important step to obtain a distribution of subjects “approximately healthy”.

## 5. Conclusions

Changes in concentrations of bone metabolism markers in pediatric and adolescent subjects present similarities. BTM concentrations during childhood do not differ in boys and girls [48]; increase with puberty [48,49], with a later increase in boys; and subsequently decrease until reaching adult concentrations, with a more rapid decrease in girls [12].

For each single BTM, we calculated RIs obtained by grouping age groups that presented concentrations with statistically insignificant differences [91] to make their clinical use more effective.

## Figures and Tables

**Table 1:** Descriptive statistics reporting for the analytes BALP, CTX, OC, PINP, and TRAcP, evaluated in full (Tot) and stratified by sex [females (F) and males (M)], the number of samples, the minimum and maximum concentrations, the calculation of the mean, the median and the standard deviation (SD) with the distribution intervals [95% confidence intervals (CI)], the evaluation of the distribution normality using the D’Agostino–Pearson test (*p* < 0.05), and the evaluation of the presence of outliers.

	BALP (μg/L)	BALP (μg/L) F	BALP (μg/L) M	CTX (ng/mL) Tot	CTX (ng/mL) F	CTX (ng/mL) M	OC	OC (ng/mL) F	OC (ng/mL) M	PINP (μg/L)	PINP (μg/L)	PINP (μg/L) M	TRAcP (U/L)	TRAcP (U/L)	TRAcP (U/L) M
	Tot						(ng/mL) Tot			Tot	F		Tot	F	
**N. subject**	202	98	104	202	98	104	202	98	104	202	98	104	202	98	104
**Minimum**	6.6	6.6	10.6	0.16	0.16	0.32	7.7	11.6	7.7	18.55	18.55	59.81	1.9	1.9	2.3
**Maximum**	186.7	163.1	186.7	2.95	2.95	2.93	193	193	192	1701	1344	1701	31	28	31
**Mean**	73.49	64.37	81.43	1.4	1.28	1.5	80.56	76.18	84.39	602.16	518.81	674.71	11.55	9.96	12.93
**95% CI**	68.15 to 78.82	56.48 to 72.25	74.41 to 88.45	1.31 to 1.48	1.14 to 1.41	1.39 to 1.61	74.77 to 86.35	67.21 to 85.14	76.81 to 91.95	550.92 to 653.39	441.87 to 595.73	608.05 to 741.36	10.65 to 12.44	8.631 to 11.286	11.75 to 14.10
**Median**	72.75	56.3	81	1.4	1.27	1.45	74.75	70.3	75.75	614.86	492.65	666.74	10.65	8.35	12.2
**95% CI**	66.60 to 81.62	43.20 to 70.98	72.73 to 86.86	1.25 to 1.50	0.92 to 1.48	1.28 to 1.60	69.75 to 81.84	54.42 to 86.49	70.53 to 84.75	557.55 to 666.57	275.85 to 604.37	626.96 to 714.16	9.65 to 11.68	6.70 to 10.00	10.90 to 13.19
**SD**	38.47	38.49	36.81	0.61	0.65	0.56	41.71	43.76	39.68	369.26	375.62	349.42	6.47	6.48	6.17
**D’Agostino–Pearson test**	reject Normality (*p* = 0.0005)			reject Normality (*p* = 0.009)			reject Normality (*p* < 0.0001)			reject Normality (*p* < 0.0001)			reject Normality (*p* < 0.0001)		
**Suspected outliers (Tukey test)**	None	None	None	None	None	None	None	None	None	None	None	None	None	None	None

**Table 2:** The measurements of BALP (μg/L), CTX (ng/mL), OC (ng/mL), PINP (μg/L), and TRAcP (U/L) stratified into 8 age groups (years) are reported. The data are grouped in their entirety and by sex (females and males); for each individual BTM, the average concentrations are reported at the 0.05 and 0.95 centiles and the maximum values (marked in grey) and minimum (marked in green).

Variable		BALP			CTX (ng/mL)			OC			PINP			TRAcP	
		(μg/L)						(ng/mL)			(μg/L)			(U/L)	
**AGE (Years)**	**Centiles**		**Mean**	**Centiles**		**Mean**	**Centiles**		**Mean**	**Centiles**		**Mean**	**Centiles**		**Mean**
	**0.05**	**0.95**		**0.05**	**0.95**		**0.05**	**0.95**		**0.05**	**0.95**		**0.05**	**0.95**	
2	12.21	154.27	71.03	0.56	2.15	0.79	32.57	129.58	48.51	49.97	1267.89	608.96	1.26	23.82	11.28
4	28.78	145.08	58.15	0.74	2.31	0.79	35.23	151.37	58.07	254.41	1169.08	457.33	3.74	23.81	10.04
6	35.06	143.44	54.19	0.79	2.45	0.83	35.28	163.69	64.21	322.94	1168.82	422.94	4.55	24.13	9.79
8	33.65	145	55.68	0.76	2.54	0.89	33.06	167.08	67.01	291.63	1216.27	462.32	4.18	24.32	10.07
10	27.14	145.42	59.14	0.65	2.56	0.96	28.93	162.08	66.57	196.53	1260.54	532	3.09	23.93	10.42
12	18.14	140.37	61.12	0.51	2.49	0.99	23.26	149.24	62.99	73.71	1250.78	588.53	1.76	22.52	10.38
14	9.24	125.5	58.13	0.34	2.3	0.98	16.4	129.1	56.35	40.78	1136.12	547.67	0.67	19.64	9.48
16	3.03	96.48	46.72	0.18	1.97	0.89	8.72	102.2	46.74	40.87	865.69	377.41	0.28	14.83	7.28
**AGE years F**															
2	40.34	203.1	81.38	0.53	3.29	1.53	34.55	204.01	84.73	268.34	2005.48	868.57	3.83	35.07	15.62
4	35.14	183.26	74.06	0.48	3.58	1.4	29.74	188.99	79.63	223.4	1784.74	780.67	3.49	31.33	13.92
6	29.93	163.41	66.74	0.43	2.99	1.28	24.93	173.98	74.52	178.46	1563.99	692.77	3.15	27.59	12.22
8	24.72	143.57	59.42	0.39	2.7	1.16	20.13	158.96	69.42	133.52	1343.25	604.87	2.81	23.85	10.52
10	19.52	123.73	52.1	0.34	2.4	1.03	15.32	143.95	64.32	88.58	1122.51	516.96	2.46	20.11	8.82
12	14.31	103.88	44.79	0.29	2.11	0.91	10.51	128.94	59.21	43.64	901.76	429.06	2.12	16.37	7.12
14	9.1	84.04	37.47	0.25	1.81	0.78	5.7	113.92	54.11	42.3	681.02	319.36	1.78	12.63	5.42
16	3.9	64.19	30.15	0.2	1.52	0.66	0.9	98.91	49	46.24	460.27	207.01	1.44	8.89	3.72
**AGE years M**															
2	58.03	132.7	67.34	1.15	2.12	1.48	49.21	129.82	60.3	596.9	1166.68	684.89	8.2	26.47	9.13
4	50.02	134.08	62.03	1.02	2.18	1.58	43.31	133.74	65.22	506.79	1151.3	622.26	7.09	25.33	9.12
6	42.02	135.46	46.72	0.89	2.25	1.68	37.41	137.67	50.13	416.68	1135.93	659.63	5.97	24.19	9.11
8	34.02	136.84	51.41	0.76	2.31	0.78	31.51	141.6	55.05	326.56	1120.55	696.99	4.86	23.06	9.1
10	26.01	138.22	56.1	0.63	2.38	0.87	25.61	145.53	59.96	236.45	1105.17	434.36	3.75	21.92	9.08
12	18.01	139.59	60.79	0.5	2.45	0.97	19.71	149.46	64.87	146.34	1089.79	471.72	2.64	20.78	9.07
14	10	140.97	65.48	0.37	2.51	1.07	13.81	133.39	60.79	56.22	1074.41	509.09	1.53	19.65	9.06
16	2	132.35	70.18	0.24	2.58	1.17	7.91	137.32	64.7	33.89	1059.03	546.46	0.41	18.51	9.05

**Table 3:** The measurements of BALP (μg/L), CTX (ng/mL), OC (ng/mL), PINP (μg/L), and TRAcP (U/L) grouped as a whole and by sex (females and males) are reported. For each BTM, lower and upper limit concentrations are reported in the age range 1–18 years and in two age subgroups based on the variability in results. The limits highlighted in blue are the proposed ones.

Variable	BALP (μg/L)			CTX (ng/mL)			OC (ng/mL)			PINP (μg/L)			TRAcP (U/L)		
	Tot	Females	Males	Tot	Females	Males	Tot	Females	Males	Tot	Females	Males	Tot	Females	Males
Age Range	1–18 y	1–18 y	1–18 y	1–18 y	1–18 y	1–18 y	1–18 y	1–18 y	1–18 y	1–18 y	1–18 y	1–18 y	1–18 y	1–18 y	1–18 y
Lower limit	14.59	12.69	15.84	0.41	0.32	0.52	17.81	14.01	22.74	63.6	57.76	86.93	2.81	2.45	3.17
Upper limit	162.28	150.71	167.64	2.9	2.9	2.91	191.92	189.68	192.63	1517.03	1316.31	1696.1	24.1	24.35	25.72
Age range	1–14 y	1–14 y	1–14 y	1–14 y	1–14 y	1–14 y	1–13 y	1–13 y	1–13 y	1–13 y	1–13 y	1–13 y	1–12 y	1–12 y	1–12 y
Lower limit	22.32	20.56	24.55	0.48	0.43	0.6	25.33	15.21	24.08	144.48	143.63	167.09	5.38	4.6	6.72
Upper limit	163.54	155.53	169.49	2.86	2.96	2.85	190.07	191.83	187.07	1617.07	1327.29	1693.04	24.11	24	23.4
Age range	15–18 y	15–18 y	15–18 y	15–18 y	15–18 y	15–18 y	14–18 y	14–18 y	14–18 y	14–18 y	14–18 y	14–18 y	13–18 y	13–18 y	13–18 y
Lower limit	7.49	6.6	16.5	0.18	0.16	0.54	14.8	12.7	23.1	37.38	48.55	83.3	2.3	2.34	1.9
Upper limit	123	60.6	130.9	2.33	1.44	2.36	153.14	109	187.1	959.22	1025	1040	23.98	21.28	30

**Table 4:** Comparison of the difference between the medians of biomarker concentrations (BALP, CTX, OC, PINP, TRAcP) in the groups of subjects stratified by age and sex (Mann–Whitney test). Statistically significant differences are highlighted in gray (*p* < 0.005).

Variable	BALP	CTX	OC	PINP	TRAcP
1–6 years vs. 7–12 years	*p* = 0.8598	*p* = 0.1124	*p* = 0.0047	*p* = 0.0032	*p* = 0.3461
1–6 years vs. 13–18 years	*p* < 0.0001	*p* < 0.0001	*p* < 0.0001	*p* = 0.2279	*p* < 0.0001
7–12 years vs. 13–18 years	*p* < 0.0001	*p* < 0.0001	*p* < 0.0001	*p* < 0.0001	*p* < 0.0001
Tot males vs. tot females	*p* = 0.0016	*p* = 0.0070	*p* = 0.1190	*p* = 0.0025	*p* = 0.0001
1–6 years females vs. 1–6 years males	*p* = 0.9673	*p* = 0.1780	*p* = 0.9026	*p* = 0.8383	*p* = 0.8171
7–12 years females vs. 7–12 years males	*p* = 0.6269	*p* = 0.8801	*p* = 0.6468	*p* = 0.9066	*p* = 0.1138
13–18 years females vs. 13–18 years males	*p* = 0.0002	*p* = 0.0009	*p* = 0.0032	*p* = 0.0004	*p* = 0.0017

**Table 5:** Multiple comparison procedures (pairwise comparison) in ANOVA test in subgroups stratified by age.

Variable	Mean Rank	Different (*p* < 0.05) from Variable
BALP 1–6 y	2.0238	BALP 13–18 y
BALP 7–12 y	2.4048	BALP 13–18 y
BALP 13–18 y	1.5714	BALP 1–6 y and BALP 7–12 y
CTX 1–6 y	1.9048	
CTX 7–12 y	2.3095	CTX 13–18 y
CTX 13–18 y	1.7857	CTX 7–12 y
OC 1–6 y	1.9048	OC 7–12 y
OC 7–12 y	2.3333	OC 1–6 y and OC 13–18 y
OC 13–18 y	1.7619	OC 7–12 y
PINP 1–6 y	2.2143	PINP 13–18 y
PINP 7–12 y	2.3571	PINP 13–18 y
PINP 13–18 y	1.4286	PINP 1–6 y and PINP 7–12 y
TRAcP 1–6 y	2.1667	TRAcP 13–18 y
TRAcP 7–12 y	2.4286	TRAcP 13–18 y
TRAcP 13–18 y	1.4048	TRAcP 1–6 y and TRAcP 7–12 y

## Data Availability

The data presented in this study are available from the corresponding author upon reasonable request.

## References

[1] Orvalho J.M., Fernandes J.C.H., Moraes Castilho R., Fernandes G.V.O. (2023). The macrophage’s role on bone remodeling and osteogenesis: A systematic review. Clin. Rev. Bone Miner. Metab..

[2] Owen R., Reilly G.C. (2018). In vitro Models of Bone Remodelling and Associated Disorders. Front. Bioeng. Biotechnol..

[3] Ladang A., Rauch F., Delvin E., Cavalier E. (2023). Bone Turnover Markers in Children: From Laboratory Challenges to Clinical Interpretation. Calcif. Tissue Int..

[4] Matsuo K., Irie N. (2008). Osteoclast-osteoblast communication. Arch. Biochem. Biophys..

[5] Eastell R., Szulc P. (2017). Use of bone turnover markers in postmenopausal osteoporosis. Lancet Diabetes Endocrinol..

[6] Parfitt A.M., Travers R., Rauch F., Glorieux F.H. (2000). Structural and cellular changes during bone growth in healthy children. Bone.

[7] Szulc P. (2018). Bone turnover: Biology and assessment tools. Best Pract. Res. Clin. Endocrinol. Metab..

[8] Rauch F. (2006). Watching bone cells at work: What we can see from bone biopsies. Pediatr. Nephrol..

[9] Diemar S.S., Lylloff L., Rønne M.S., Møllehave L.T., Heidemann M., Thuesen B.H., Johannesen J., Schou A.J., Husby S., Wedderkopp N. (2021). Reference intervals in Danish children and adolescents for bone turnover markers carboxy-terminal cross-linked telopeptide of type I collagen (β-CTX), pro-collagen type I N-terminal propeptide (PINP), osteocalcin (OC) and bone-specific alkaline phosphatase (bone ALP). Bone.

[10] Callegari E.T., Gorelik A., Garland S.M., Chiang C.Y., Wark J.D. (2017). Bone turnover marker reference intervals in young Females. Ann. Clin. Biochem..

[11] Glover S.J., Garnero P., Naylor K., Rogers A., Eastell R. (2008). Establishing a reference range for bone turnover markers in young, healthy women. Bone.

[12] Zürcher S.J., Borter N., Kränzlin M., Neyer P., Meyer U., Rizzoli R., Kriemler S. (2020). Relationship between bone mineral content and bone turnover markers, sex hormones and calciotropic hormones in pre- and early pubertal children. Osteoporos. Int..

[13] Michou L., Orcel P. (2016). The changing countenance of Paget’s Disease of bone. Jt. Bone Spine.

[14] Yavropoulou M.P., Tomos K., Tsekmekidou X., Anastasiou O., Zebekakis P., Karamouzis M., Gotzamani-Psarrakou A., Chassapopoulou E., Chalkia P., Yovos J.G. (2011). Response of biochemical markers of bone turnover to oral glucose load in diseases that affect bone metabolism. Eur. J. Endocrinol..

[15] Hu T., Yang Q., Xu J., Zhang Z., He N., Du Y. (2015). Role of β-isomerized C-terminal telopeptides (β-CTx) and total procollagen type 1 amino-terminal propeptide (tP1NP) as osteosarcoma biomarkers. Int. J. Clin. Exp. Med..

[16] Garnero P. (2014). New developments in biological markers of bone metabolism in osteoporosis. Bone.

[17] Gayretli Aydin Z.G., Bideci A., Emeksiz H.C., Çelik N., Döğer E., Bukan N., Yildiz U., Camurdan O.M., Cinaz P. (2015). Assessment of bone turnover markers and bone mineral density in normal short boys. J. Pediatr. Endocrinol. Metab..

[18] Barber L.A., Abbott C., Nakhate V., Do A.N.D., Blissett A.R., Marini J.C. (2019). Longitudinal growth curves for children with classical osteogenesis imperfecta (types III and IV) caused by structural pathogenic variants in type I collagen. Genet. Med..

[19] Khadka B., Tiwari M.L., Gautam R., Timalsina B., Pathak N.P., Kharel K., Sharma S., Acharya D. (2018). Correlates of Biochemical Markers of Bone turnover among Post-Menopausal Women. JNMA J. Nepal. Med. Assoc..

[20] Huang Y., Eapen E., Steele S., Grey V. (2011). Establishment of reference intervals for bone markers in children and adolescents. Clin. Biochem..

[21] Friedberg R.C., Souers R., Wagar E.A., Stankovic A.K., Valenstein P.N., College of American Pathologists (2007). The origin of reference intervals. Arch. Pathol. Lab. Med..

[22] Özçürümez M.K., Haeckel R. (2018). Biological variables influencing the estimation of reference limits. Scand. J. Clin. Lab. Investig..

[23] D’Amato G., Brescia V., Fontana A., Natale M.P., Lovero R., Varraso L., Di Serio F., Simonetti S., Muggeo P., Faienza M.F. (2024). Biomarkers and Biochemical Indicators to Evaluate Bone Metabolism in Preterm Neonates. Biomedicines.

[24] Nizet A., Cavalier E., Stenvinkel P., Haarhaus M., Magnusson P. (2020). Bone alkaline phosphatase: An important biomarker in chronic kidney disease—Mineral and bone disorder. Clin. Chim. Acta.

[25] Van Hoof V.O., De Broe M.E. (1994). Interpretation and clinical significance of alkaline phosphatase isoenzyme patterns. Crit. Rev. Clin. Lab. Sci..

[26] Ahmed F., Gibbons S.M. (2015). Bone-specific alkaline phosphatase by immunoassay or electrophoresis: Their use in clinical practice. J. Clin. Pathol..

[27] Rosalki S.B., Foo A.Y. (1984). Two new methods for separating and quantifying bone and liver alkaline phosphatase isoenzymes in plasma. Clin. Chem..

[28] Price C.P., Mitchell C.A., Moriarty J., Gray M., Noonan K. (1995). Mass versus activity: Validation of an immunometric assay for bone alkaline phosphatase in serum. Ann. Clin. Biochem..

[29] Farley J.R., Hall S.L., Ilacas D., Orcutt C., Miller B.E., Hill C.S., Baylink D.J. (1994). Quantification of skeletal alkaline phosphatase in osteoporotic serum by wheat germ agglutinin precipitation, heat inactivation, and a two-site immunoradiometric assay. Clin. Chem..

[30] Moss D.W., Whitby L.G. (1975). A simplified heat-inactivation method for investigating alkaline phosphatase isoenzymes in serum. Clin. Chim. Acta.

[31] Cavalier E., Souberbielle J.C., Gadisseur R., Dubois B., Krzesinski J.M., Delanaye P. (2014). Inter-method variability in bone alkaline phosphatase measurement: Clinical impact on the management of dialysis patients. Clin. Biochem..

[32] Milinković N., Sarić-Matutinović M., Pejanović S., Ignjatović S. (2020). Comparison between bone alkaline phosphatase immunoassay and electrophoresis technique in hemodialysis patients. J. Med. Biochem..

[33] Christensen G.L., Halgreen J.R., Milenkovski M., Köse A., Quardon N., Jørgensen N.R. (2019). Bone turnover markers are differentially affected by pre-analytical handling. Osteoporos. Int..

[34] Cavalier E., Rozet E., Carlisi A., Bekaert A.C., Rousselle O., Hubert P., Chapelle J.P., Delanaye P. (2010). Analytical validation of serum bone alkaline phosphatase (BAP OSTASE) on Liaison. Clin. Chem. Lab. Med..

[35] Garnero P., Delmas P.D. (1993). Assessment of the serum levels of bone alkaline phosphatase with a new immunoradiometric assay in patients with metabolic bone disease. J. Clin. Endocrinol. Metab..

[36] Withold W., Rick W. (1994). Evaluation of an immunoradiometric assay for bone alkaline phosphatase mass concentration in human sera. Eur. J. Clin. Chem. Clin. Biochem..

[37] Chubb S.A., Mandelt C.D., Vasikaran S.D. (2015). Comparison of results from commercial assays for plasma CTX: The need for harmonization. Clin. Biochem..

[38] Lee A.J., Hodges S., Eastell R. (2000). Measurement of osteocalcin. Ann. Clin. Biochem..

[39] Garnero P., Grimaux M., Demiaux B., Preaudat C., Seguin P., Delmas P.D. (1992). Measurement of serum osteocalcin with a human-specific two-site immunoradiometric assay. J. Bone Miner. Res..

[40] Schmidt-Gayk H., Spanuth E., Kötting J., Bartl R., Felsenberg D., Pfeilschifter J., Raue F., Roth H.J. (2004). Performance evaluation of automated assays for beta-CrossLaps, N-MID-Osteocalcin and intact parathyroid hormone (BIOROSE Multicenter Study). Clin. Chem. Lab. Med..

[41] Rosenquist C., Qvist P., Bjarnason N., Christiansen C. (1995). Measurement of a more stable region of osteocalcin in serum by ELISA with two monoclonal antibodies. Clin. Chem..

[42] Koivula M.K., Ruotsalainen V., Björkman M., Nurmenniemi S., Ikäheimo R., Savolainen K., Sorva A., Risteli J. (2010). Difference between total and intact assays for N-terminal propeptide of type I procollagen reflects degradation of pN-collagen rather than denaturation of intact propeptide. Ann. Clin. Biochem..

[43] Cavalier E., Lukas P., Carlisi A., Gadisseur R., Delanaye P. (2013). Aminoterminal propeptide of type I procollagen (PINP) in chronic kidney disease patients: The assay matters. Clin. Chim. Acta.

[44] Schini M., Vilaca T., Gossiel F., Salam S., Eastell R. (2023). Bone Turnover Markers: Basic Biology to Clinical Applications. Endocr. Rev..

[45] Cavalier E., Lukas P., Delanaye P. (2021). Analytical evaluation of the Nittobo Medical tartrate resistant acid phosphatase isoform 5b (TRACP-5b) EIA and comparison with IDS iSYS in different clinically defined populations. Clin. Chem. Lab. Med..

[46] Brescia V., Fontana A., Lovero R., Capobianco C., Marsico S.V., De Chirico T., Pinto C., Varraso L., Cazzolla A.P., Di Serio F. (2022). Determination of iFGF23 Upper Reference Limits (URL) in healthy pediatric population, for its better correct use. Front. Endocrinol..

[47] Blumsohn A., Hannon R.A., Wrate R., Barton J., al-Dehaimi A.W., Colwell A., Eastell R. (1994). Biochemical markers of bone turnover in girls during puberty. Clin. Endocrinol..

[48] Rauchenzauner M., Schmid A., Heinz-Erian P., Kapelari K., Falkensammer G., Griesmacher A., Finkenstedt G., Högler W. (2007). Sex- and age-specific reference curves for serum markers of bone turnover in healthy children from 2 months to 18 years. J. Clin. Endocrinol. Metab..

[49] van der Sluis I.M., Hop W.C., van Leeuwen J.P., Pols H.A., de Muinck Keizer-Schrama S.M. (2002). A cross-sectional study on biochemical parameters of bone turnover and vitamin d metabolites in healthy dutch children and young adults. Horm. Res..

[50] Yang L., Grey V. (2006). Pediatric reference intervals for bone markers. Clin. Biochem..

[51] Jones G.R.D., Haeckel R., Loh T.P., Sikaris K., Streichert T., Katayev A., Barth J.H., Ozarda Y., IFCC Committee on Reference Intervals and Decision Limits (2018). Indirect methods for reference interval determination—Review and recommendations. Clin. Chem. Lab. Med..

[52] Finkelstein J.S., Sowers M., Greendale G.A., Lee M.L., Neer R.M., Cauley J.A., Ettinger B. (2002). Ethnic variation in bone turnover in pre- and early perimenopausal women: Effects of anthropometric and lifestyle factors. J. Clin. Endocrinol. Metab..

[53] Gombos Császár G., Bajsz V., Sió E., Steinhausz Tóth V., Schmidt B., Szekeres L., Kránicz J. (2014). The direct effect of specific training and walking on bone metabolic markers in young adults with peak bone mass. Acta Physiol. Hung..

[54] Redmond J., Fulford A.J., Jarjou L., Zhou B., Prentice A., Schoenmakers I. (2016). Diurnal Rhythms of Bone Turnover Markers in Three Ethnic Groups. J. Clin. Endocrinol. Metab..

[55] Seibel M.J. (2005). Biochemical markers of bone turnover: Part I: Biochemistry and variability. Clin. Biochem. Rev..

[56] Price C.P. (1993). Multiple forms of human serum alkaline phosphatase: Detection and quantitation. Ann. Clin. Biochem..

[57] Blumsohn A., Hannon R.A., Eastell R. (1995). Apparent instability of osteocalcin in serum as measured with different commercially available immunoassays. Clin. Chem..

[58] Halleen J.M., Tiitinen S.L., Ylipahkala H., Fagerlund K.M., Vaananen H.K. (2006). Tartrate-resistant acid phosphatase 5b (TRACP 5b) as a marker of bone resorption. Clin. Lab..

[59] Garnero P., Vergnaud P., Hoyle N. (2008). Evaluation of a fully automated serum assay for total N-terminal propeptide of type I collagen in postmenopausal osteoporosis. Clin. Chem..

[60] Morovat A., Catchpole A., Meurisse A., Carlisi A., Bekaert A.C., Rousselle O., Paddon M., James T., Cavalier E. (2013). IDS iSYS automated intact procollagen-1-N-terminus pro-peptide assay: Method evaluation and reference intervals in adults and children. Clin. Chem. Lab. Med..

[61] Okabe R., Nakatsuka K., Inaba M., Miki T., Naka H., Masaki H., Moriguchi A., Nishizawa Y. (2001). Clinical evaluation of the Elecsys beta-CrossLaps serum assay, a new assay for degradation products of type I collagen C-tlopeptides. Clin. Chem..

[62] Naylor K.E., Iqbal P., Fledelius C., Fraser R.B., Eastell R. (2000). The effect of pregnancy on bone density and bone turnover. J. Bone Miner. Res..

[63] Ivaska K.K., Gerdhem P., Akesson K., Garnero P., Obrant K.J. (2007). Effect of fracture on bone turnover markers: A longitudinal study comparing marker levels before and after injury in 113 elderly women. J. Bone Miner. Res..

[64] McAssey K.L., Grey V., Dietzen D.J., Bennett M.J., Wong E.C.C. (2010). Disorders of calcium and phosphate metabolism in infants and children. Biochemical and Molecular Basis of Pediatric Disease.

[65] Holick M.F., Binkley N.C., Bischoff-Ferrari H.A., Gordon C.M., Hanley D.A., Heaney R.P., Murad M.H., Weaver C.M., Endocrine Society (2011). Evaluation, treatment, and prevention of vitamin D deficiency: An Endocrine Society clinical practice guideline. J. Clin. Endocrinol. Metab..

[66] Sempos C.T., Heijboer A.C., Bikle D.D., Bollerslev J., Bouillon R., Brannon P.M., DeLuca H.F., Jones G., Munns C.F., Bilezikian J.P. (2018). Vitamin D assays and the definition of hypovitaminosis D: Results from the First International Conference on Controversies in Vitamin D. Br. J. Clin. Pharmacol..

[67] Vierucci F., Del Pistoia M., Fanos M., Gori M., Carlone G., Erba P., Massimetti G., Federico G., Saggese G. (2013). Vitamin D status and predictors of hypovitaminosis D in Italian children and adolescents: A cross-sectional study. Eur. J. Pediatr..

[68] Hansen L., Tjønneland A., Køster B., Brot C., Andersen R., Cohen A.S., Frederiksen K., Olsen A. (2018). Vitamin D Status and Seasonal Variation among Danish Children and Adults: A Descriptive Study. Nutrients.

[69] Vierucci F., Del Pistoia M., Fanos M., Erba P., Saggese G. (2014). Prevalence of hypovitaminosis D and predictors of vitamin D status in Italian healthy adolescents. Ital. J. Pediatr..

[70] Eloi M., Horvath D.V., Szejnfeld V.L., Ortega J.C., Rocha D.A., Szejnfeld J., Castro C.H. (2016). Vitamin D deficiency and seasonal variation over the years in São Paulo, Brazil. Osteoporos. Int..

[71] Sikaris K.A. (2022). The Role of Mimicry in Defining Statistical Health. Clin. Chem..

[72] Ladang A., Rousselle O., Huyghebaert L., Bekaert A.C., Kovacs S., Le Goff C., Cavalier E. (2022). Parathormone, bone alkaline phosphatase and 25-hydroxyvitamin D status in a large cohort of 1200 children and teenagers. Acta Clin. Belg..

[73] Fischer D.C., Mischek A., Wolf S., Rahn A., Salweski B., Kundt G., Haffner D. (2012). Paediatric reference values for the C-terminal fragment of fibroblast-growth factor-23, sclerostin, bone-specific alkaline phosphatase and isoform 5b of tartrate-resistant acid phosphatase. Ann. Clin. Biochem..

[74] Colantonio D.A., Kyriakopoulou L., Chan M.K., Daly C.H., Brinc D., Venner A.A., Pasic M.D., Armbruster D., Adeli K. (2012). Closing the gaps in pediatric laboratory reference intervals: A CALIPER database of 40 biochemical markers in a healthy and multiethnic population of children. Clin. Chem..

[75] Choi J.S., Park I., Lee S.J., Ju H.J., Lee H., Kim J. (2019). Serum Procollagen Type I N-Terminal Propeptide and Osteocalcin Levels in Korean Children and Adolescents. Yonsei Med. J..

[76] Cavalier E., Eastell R., Rye Jørgensen N., Makris K., Tournis S., Vasikaran S., Kanis J.A., Cooper C., Pottel H., Morris H.A. (2019). A multicenter study to evaluate harmonization of assays for N-terminal propeptide of type I procollagen (PINP): A report from the IFCC-IOF Joint Committee for Bone Metabolism. Clin. Chem. Lab. Med..

[77] Bayer M. (2014). Reference values of osteocalcin and procollagen type I N-propeptide plasma levels in a healthy Central European population aged 0-18 years. Osteoporos. Int..

[78] Wyness S.P., Roberts W.L., Straseski J.A. (2013). Pediatric reference intervals for four serum bone markers using two automated immunoassays. Clin. Chim. Acta.

[79] Vasikaran S.D., Bhattoa H.P., Eastell R., Heijboer A.C., Jørgensen N.R., Makris K., Ulmer C., Kanis J.A., Cooper C., Silverman S. (2020). Harmonization of commercial assays for PINP; the way forward. Osteoporos. Int..

[80] Dioguardi M., Spirito F., Sovereto D., Alovisi M., Aiuto R., Garcovich D., Crincoli V., Laino L., Cazzolla A.P., Caloro G.A. (2022). The Prognostic Role of miR-31 in Head and Neck Squamous Cell Carcinoma: Systematic Review and Meta-Analysis with Trial Sequential Analysis. Int. J. Environ. Res. Public Health.

[81] Geserick M., Vogel M., Eckelt F., Schlingmann M., Hiemisch A., Baber R., Thiery J., Körner A., Kiess W., Kratzsch J. (2020). Children and adolescents with obesity have reduced serum bone turnover markers and 25-hydroxyvitamin D but increased parathyroid hormone concentrations—Results derived from new pediatric reference ranges. Bone.

[82] Herrmann D., Intemann T., Lauria F., Mårild S., Molnár D., Moreno L.A., Sioen I., Tornaritis M., Veidebaum T., Pigeot I. (2014). Reference values of bone stiffness index and C-terminal telopeptide in healthy European children. Int. J. Obes..

[83] de Melo V.C.P., Ferreira P.R.S., Ricardi L.O., Batista M.C., França C.N., Ferreira C.E.D.S. (2018). Definition of reference ranges for β-isomerized carboxy-terminal telopeptide collagen type I for children and adolescents. J. Pediatr. Endocrinol. Metab..

[84] Thiering E., Brüske I., Kratzsch J., Hofbauer L.C., Berdel D., von Berg A., Lehmann I., Hoffmann B., Bauer C.P., Koletzko S. (2015). Associations between serum 25-hydroxyvitamin D and bone turnover markers in a population based sample of German children. Sci. Rep..

[85] Dimitri P., Wales J.K., Bishop N. (2011). Adipokines, bone-derived factors and bone turnover in obese children; evidence for altered fat-bone signalling resulting in reduced bone mass. Bone.

[86] Crincoli V., Ballini A., Fatone L., Di Bisceglie M.B., Nardi G.M., Grassi F.R. (2016). Cytokine genotype distribution in patients with periodontal disease and rheumatoid arthritis or diabetes mellitus. J. Biol. Regul. Homeost. Agents.

[87] Chen C.J., Chao T.Y., Janckila A.J., Cheng S.N., Ku C.H., Chu D.M. (2005). Evaluation of the activity of tartrate-resistant acid phosphatase isoform 5b in normal Chinese children—A novel marker for bone growth. J. Pediatr. Endocrinol. Metab..

[88] Greenblatt M.B., Tsai J.N., Wein M.N. (2017). Bone Turnover Markers in the Diagnosis and Monitoring of Metabolic Bone Disease. Clin. Chem..

[89] Cavalier E., Huyghebaert L., Rousselle O., Bekaert A.C., Kovacs S., Vranken L., Peeters S., Le Goff C., Ladang A. (2020). Simultaneous measurement of 25(OH)-vitamin D and 24,25(OH)2-vitamin D to define cut-offs for CYP24A1 mutation and vitamin D deficiency in a population of 1200 young subjects. Clin. Chem. Lab. Med..

[90] CLSI (2008). Defining, Establishing, and Verifying Reference Intervals in the Clinical Laboratory; Approved Guidelines.

[91] Henny J., Arnaud J., Giroud C., Vassault A. (2010). Determination and verification of reference intervals. Ann. Biol. Clin..

